# Molecular changes in peripheral blood involving osteoarthritic joint remodelling

**DOI:** 10.1111/joor.12810

**Published:** 2019-05-11

**Authors:** Hong‐Yun Zhang, Qian Liu, Jin‐Qiang Liu, Jing Wang, Hong‐Xu Yang, Xiao‐Jie Xu, Mian‐Jiao Xie, Xiao‐Dong Liu, Shi‐Bin Yu, Mian Zhang, Lei Lu, Jing Zhang, Mei‐Qing Wang

**Affiliations:** ^1^ State Key Laboratory of Military Stomatology, National Clinical Research Centre for Oral Disease & Shaanxi International Joint Research Centre for Oral Diseases Department of Oral Anatomy and Physiology and TMD, School of Stomatology The Fourth Military Medical University Xi'an China; ^2^ School of Stomatology The Jiamusi University Jiamusi China; ^3^ Department of Burns and Cutaneous Surgery, Xijing Hospital The Fourth Military Medical University Xi'an China; ^4^ The Third Affiliated Hospital of Xinxiang Medical University Xinxiang China

**Keywords:** biomarkers, osteoarthritis, temporomandibular joint

## Abstract

Biomarkers of temporomandibular joint (TMJ) osteoarthritis (OA) remain unknown. The objective was to detect whether molecular biomarkers from peripheral blood leucocytes (PBLs) engage in TMJ OA lesions. Thirty‐four six‐week‐old Sprague Dawley rats were used. The top upregulated gene ontology categories and gene‐fold changes in PBLs were detected by a microarray analysis comparing rats that received 20‐week unilateral anterior crossbite (UAC) treatment with age‐matched controls (n = 4). Twenty weeks of UAC treatment had been reported to induce TMJ OA‐like lesions. The other twenty‐four rats were randomly placed in the UAC and control groups at 12‐ and 20‐week time points (n = 6). The mRNA expression levels of the selected biomarkers derived from the microarray analysis and their protein expression in the alveolar bone and TMJ were detected. The microarray analysis indicated that the three most highly involved genes in PBLs were *Egr1*, *Ephx1* and* Il10*, which were confirmed by real‐time PCR detection. The increased protein expression levels of the three detected molecules were demonstrated in cartilage and subchondral bone (*P* < 0.05), and increased levels of EPHX1 were reported in discs (*P* < 0.05); however, increased levels were not present in the alveolar bone. Immunohistochemistry revealed the increased distribution of EGR1‐positive, EXPH1‐positive and IL10‐positive cells predominantly in the osteochondral interface, with EXPH1 also present in TMJ discs. In conclusion, the increased mRNA expression of *Egr1, Ephx1* and* Il10* in PBLs may serve as potential biomarkers for developed osteoarthritic lesions relating to osteochondral interface hardness changes induced by dental biomechanical stimulation.

## 1 BACKGROUND

Osteoarthritis (OA) is a widespread joint problem that is closely associated with biomechanical factors.^1^ The temporomandibular joint (TMJ), which is biomechanically associated with dental occlusions, is often insulted by osteoarthritis.^2^ The diagnosis of OA is often based on osseous changes on TMJ images, such as those revealed by X‐ray and computerised tomographic (CT) scanning.^3^, ^4^ Blood cells flow all around the body and respond to internal and external signals; therefore, changes in TMJs should be reflected in the cells in the peripheral blood.^5^ Whether the OA changes in the TMJs induced by dental occlusion can be indicated by biological markers in peripheral blood leucocytes (PBLs) is an area of interest.

Currently, the microarray technique has advanced to be able to detect the relative expression levels of potentially interesting genes in a single experiment.^5^, ^6^ This technology has been used in studies on pain‐related diseases including oro‐facial pain^7^ and in the biological activities of chondrocytes, such as those observed to respond to an in vitro stimulator and in comparisons of samples from healthy and osteoarthritic joints.^8^, ^9^ Thus, there is a possibility of gene detection in PBLs for the TMJ OA lesions; however, this possibility remains unexplored.

Animal studies are generally accepted for human disease investigations. Many studies have been performed using animal models, including mice and rats, to alter dental occlusions to study TMJ OA.^10^, ^11^, ^12^ Based on a gene array analysis, Appleton et al indicated that the gene expression changes in a surgically developed rat OA model were similar to those found in human OA.^13^ Recently, we developed a prosthetic unilateral anterior crossbite (UAC) rodent model in which typical OA‐like lesions were observed in TMJs, such as chondrocyte death, cartilage matrix loss, osteochondral interface stiffening and abnormal reparative bone turnover.^14^, ^15^, ^16^, ^17^, ^18^ In the present study, we first detected molecular changes in the PBLs of UAC rats. We then described the significance of the molecular changes disclosed in the PBLs of the UAC rats in the related tissues, including the alveolar bone, TMJ disc, mandibular condylar cartilage and subchondral bone. The purpose of this study was to “fingerprint” the molecular changes in PBLs that may be linked to severe OA lesions in TMJs; these findings will bring new insights regarding clinical biomarker detection and the evaluation of TMJ OA lesions.

## 2 MATERIALS AND METHODS

The animal studies were approved by the Laboratory Animal Care and Welfare Committee, School of Stomatology, Fourth Military Medical University for animal research (2015‐033).

### 2.1 Animals and UAC operation

As we previously reported, there were slight gross morphological changes in the TMJ condyle heads of UAC‐treated Sprague Dawley (SD) rats at 12 weeks and deformations such as twisted shapes at 20 weeks. Significant mineralisation was exhibited at 12 and 20 weeks after UAC operation, which was displayed as osteochondral interface stiffening.^16^, ^19^, ^20^ As a result, we used the 20‐week group for the PBL mRNA microarray detections and the 12‐ and 20‐week groups to detect the TMJ and alveolar tissues for verification. There was an 8‐weeks gap between each time points.

Thirty‐two 6‐week‐old female SD rats (150‐160 g) were included in this study. Eight of the rats were used for the gene array analysis and randomly assigned to the sham‐operated control (Con_Gene) or UAC_Gene groups (n = 4). These eight rats were sacrificed at the end of 20 weeks after the UAC operation. The remaining 24 rats were randomly assigned to four groups, that is, two sham‐operated groups (CON) and two UAC groups (n = 6) that were sacrificed at the end of 12 and 20 weeks after the UAC operation, respectively.

UAC was created as previously described by our laboratory.^14^, ^15^, ^16^ Briefly, a straight metal tube (ShinvaAnde, Shandong, China) and a curved metal tube (ShinvaAnde, Shandong, China) with an angle of 135° to the labial side at the upper end were bonded with zinc phosphate cement (Shanghai Dental Instrument Factory, Shanghai, China) to the left maxillary and mandibular incisor, respectively, to create an experimental crossbite relationship between the left side incisors. The rats in the control group underwent similar procedures but without bonding of the metal tubes. All rats were housed at 23°C with 30‐40% relative humidity and normal 12 hours light and 12 hours dark cycles. They were fed with standard cylindrically shaped pressed food pellets.

### 2.2 Blood sampling and processing for gene analysis

An expression analysis with the mRNA microarrays was performed on PBLs from the Con_Gene and UAC_Gene groups as an initial screening tool and to guide the subsequent gene expression studies. The rats were euthanised with an intraperitoneal injection of 1% pentobarbital (3 mL/kg body weight). Immediately, approximately 5 mL of whole blood was collected from the heart of each rat in each group.

Each blood sample was maintained in an ethylenediaminetetraacetic acid (EDTA) centrifuge tube. The PBLs were separated and collected from the whole blood by a kit (WBC1083, TBDscience, Tianjin, China) following the manufacturer's instructions. Then, the PBL samples from the Con_Gene and UAC_Gene groups were lysed in TRIpure reagent (Roche, Basel, Switzerland) at −80°C and delivered with dry ice to CapitalBio Technology Company (China) for RNA quality, purity and microarray processing. The whole‐genome microarray analysis of the PBLs was conducted in the same laboratory by one person following standard operating protocols to minimise non‐biological technical bias.

Differentially expressed genes were organised into hierarchical categories using Gene Ontology (GO; http://www.geneontology.org/). Significances were analysed with the Kyoto Encyclopedia of Genes and Genomes (KEGG) database (http://www.genome.jp/kegg/). Details regarding these GO and pathway analyses are described in the Supplemental methods.

The other 24 PBL samples from the two CON and two UAC groups were lysed in TRIpure reagent (Roche, Basel, Switzerland) for real‐time PCR detection.

### 2.3 Real‐time PCR

To verify the RNA sequencing data of the Con_Gene and UAC_Gene groups, real‐time PCR was performed on the PBL RNA from the two CON and two UAC groups. Prime Script RT Master Mix Perfect Real‐time (TaKaRa, Dalian, China) was used for the reverse transcription of mRNA into cDNA. The following rat primers were employed: early growth response 1 (*Egr1*): GAACAACCCTACGAGCACCTG (forward), GCCACAAAGTGTTGCCACTG (reverse); epoxide hydrolase 1 (*Ephx1*): GCCAGGGTCAAAGCCATCA (forward), ATGCCCGGAACCTATCTATCCTC (reverse); interleukin 10 (*Il10*): CAGACCCACATGCTCCGAGA (forward), CAAGGCTTGGCAACCCAAGTA (reverse); and *Gapdh* (housekeeping): GGCACAGTCAAGGCTGAGAATG (forward), ATGGTGGTGAAGACGCCAGTA (reverse). Real‐time PCR was conducted in a thermo‐cycler (Bio‐Rad Laboratories, Hercules, CA). All data were normalised to the GADPH internal standard and calculated using the 2ΔΔ‐CT method. Three observations were performed for each sample, and the mean value was used to represent the expression level of the respective sample.

### 2.4 Alveolar bone and TMJ samples

There were no differences in the histomorphology or molecular properties of the two sides according to our previous study.^14^ The mandibular alveolar bone, TMJ discs, condyle cartilage and subchondral bone were rapidly removed in each UAC and CON group. The right side of the mandibular alveolar bones and TMJ tissues were used for protein preparation (n = 6). On the left side, the TMJ blocks were prepared for immunohistochemistry (IHC) staining as previously reported (n = 6).^16^

### 2.5 Histomorphometric measurements

Haematoxylin and eosin (HE) staining was performed as we previously reported.^15^ Images were obtained with a Leica DFC490 system.

For IHC staining, a standard, three‐step, avidin‐biotin complex staining procedure was performed with anti‐EGR1 (1:100, 4153, Cell Signalling Technology, MA, USA), anti‐Exph1 (1:200, orb340819, Biorbyt LLC, CA, USA) and anti‐IL10 (1:200, ab192271, abcam, OR, USA) primary antibodies to detect changes in lower anterior alveolar bone and TMJs including disc, condylar cartilage and subchondral bone to detect the location of the three proteins.

### 2.6 Western blot

A BCA Protein Assay Kit (23225, Thermo Scientific Pierce, IL, USA) was used for protein quantification. Rabbit monoclonal antibody anti‐EGR1 (1:1000; 4153, Cell Signalling Technology, MA, USA), rabbit polyclonal antibody anti‐Exph1 (1:500; orb340819, Biobyt, CA, USA), mouse monoclonal antibody anti‐IL10 (60269‐1‐Ig, proteintech, IL, USA), rabbit polyclonal antibody anti‐β‐actin (1:2000, NC021, Zhuangzhibio, Shaanxi, China), horseradish peroxidase‐conjugated secondary antibody (A0216, Beyotime Biotechnology, Shanghai, China) and enhanced chemiluminescence detection (32132, Thermo Scientific Pierce, IL, USA) were employed.

### 2.7 Statistical analysis

The statistical analysis was performed using a two‐way ANOVA test of variance after confirming the normality and homoscedasticity of the data sets in the two CON and UAC groups. When significant differences were identified, post hoc Student‐Newman‐Keuls tests were used to identify between‐group differences. For all analyses, α = 0.05.

## 3 RESULTS

### 3.1 Microarray detection of Rat PBLs

**Figure 1:**
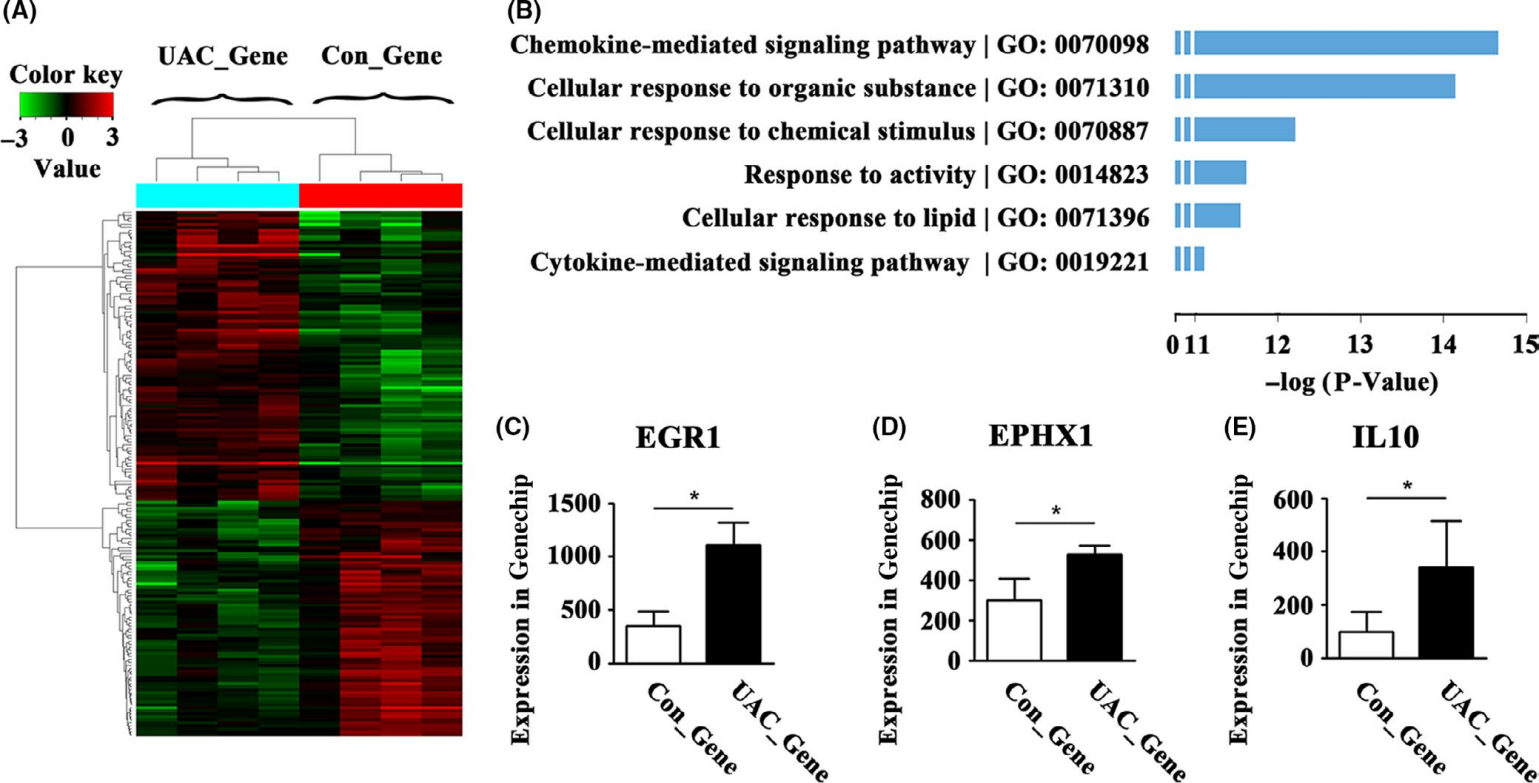
The GO database analysis of PBL mRNA of the Con_Gene and UAC_Gene groups (n = 4). (A) Heatmap comparison of normalised signals (log2) in the Con_Gene and UAC_Gene groups. (B) The top six upregulated GO categories changes in −log. (C‐E) The raw signals in the GeneChip of *Egr1*, *Ephx1* and *Il10*. GO = gene ontology; UAC = unilateral anterior crossbite; Con = control; *P < 0.05

**Figure 2:**
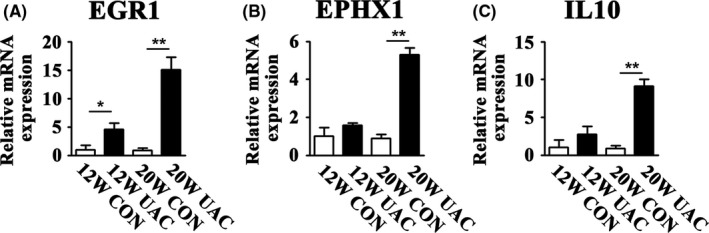
Real‐time PCR detection in the PBL mRNA focusing on the three selected upregulated genes in the top six upregulated GO categories, that is, *Egr1, Ephx1* and *Il10* (n = 6). 12W = 12 weeks, 20W = 20 weeks; CON = control group; UAC = unilateral anterior crossbite group; *P < 0.05; **P < 0.01

A total of 18 702 probes were tested. There were 96 upregulated and 78 downregulated genes in the PBLs of the UAC_Gene group, which was a change of more than 2‐fold compared to that of the Con_Gene group (Figure 1A). Analysis by the GO database showed that the top six GO categories were significantly upregulated in the UAC_Gene group, including the chemokine‐mediated signalling pathway, cellular response to organic substances, cellular response to chemical stimuli, response to activity, cellular response to lipids and the cytokine‐mediated signalling pathway. Of these top six upregulated GO categories (Figure 1B), with a *P* value <0.05 and fold changes >2, three genes related to bone and cartilage were selected. The three genes were *Egr1*,* Ephx1* and *Il10*, which were involved in three of the top six upregulated GO categories, including cellular response to organic substances, cellular response to chemical stimuli and cellular response to lipids. The expression in the GeneChip of these three genes increased, respectively (Figure 1C‐E). These upregulations were confirmed by a real‐time PCR assay for PBLs from UAC rats at 12 and 20 weeks. Real‐time PCR data indicated that the mRNA expression level of *Egr1* had the earliest fold change in the UAC group at 12 weeks, whereas all three genes were increased at 20 weeks (Figure 2, *P* < 0.05).

### 3.2 Protein expression detection for the three selected molecules

**Figure 3:**
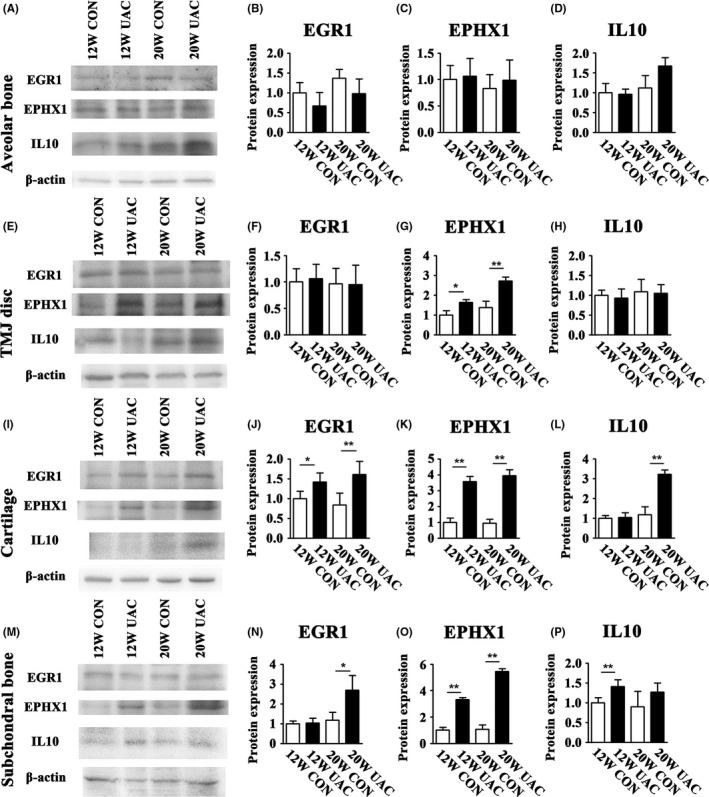
Comparison between the UAC and age‐matched control groups for the protein expression levels of EGR1, EPHX1 and IL10 in the alveolar bone, TMJ disc, condylar cartilage and subchondral bone using a Western blot assay (n = 6). 12W = 12 weeks, 20W = 20 weeks; CON = control group; UAC = unilateral anterior crossbite group; *P < 0.05; **P < 0.01

The expression levels of the three gene‐encoded proteins were increased in the TMJ tissues. The protein expression levels of EGR1, EPHX1 and IL10 were higher in the condylar cartilage and the subchondral bone of the UAC group at both time points (*P* < 0.05, except for EGR1 at 12 weeks in the subchondral bone and IL10 at 12 weeks in subchondral bone and 20 weeks in the cartilage), whereas that of EPHX1 was higher in the discs at both 12 and 20 weeks (*P* < 0.05). No such differences were noticed in alveolar bone tissues (Figure 3).

### 3.3 EGR1, EPHX1 and IL10 locations in the TMJ

**Figure 4:**
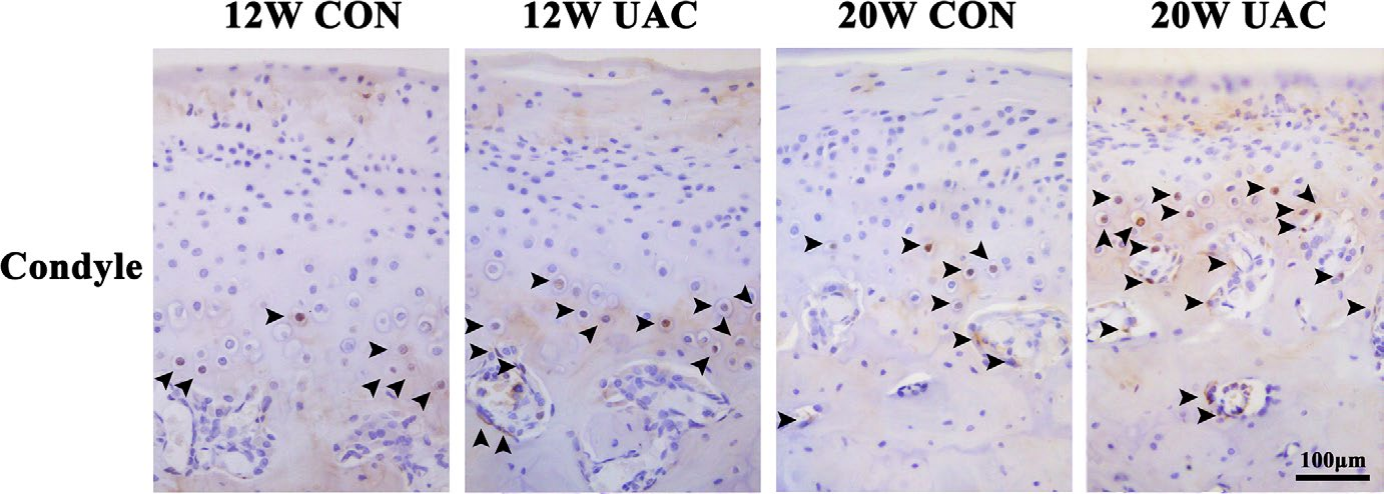
Representative immunohistochemistry images from the UAC and age‐matched control groups for the expression of EGR1 in the mandibular condyle at 12 and 20 weeks (Bar = 100 μm). 12W = 12 weeks, 20W = 20 weeks; CON = control group; UAC = unilateral anterior crossbite group

**Figure 5:**
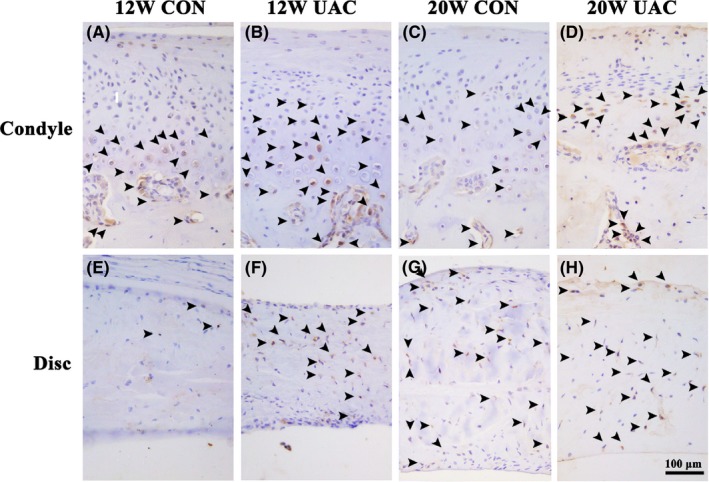
Representative immunohistochemistry images from the UAC and age‐matched control groups for the expression of EPHX1 in the mandibular condyle and disc at 12 and 20 weeks (Bar = 100 μm). 12W = 12 weeks, 20W = 20 weeks; CON = control group; UAC = unilateral anterior crossbite group

**Figure 6:**
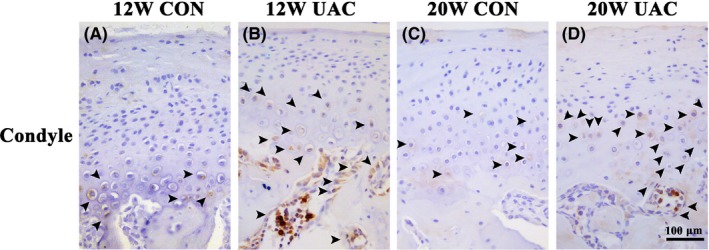
Representative immunohistochemistry images from the UAC and age‐matched control groups for the expression of IL10 in the mandibular condyle at 12 and 20 weeks (Bar = 100 μm). 12W = 12 weeks, 20W = 20 weeks; CON = control group; UAC = unilateral anterior crossbite group

In the CON groups, a small number of EGR1‐positive, EXPH1‐positive or IL10‐positive cells were scattered in the osteochondral interface, and the margin of the subchondral trabecular bone was revealed by IHC staining. In the UAC groups, EGR1‐positive, EXPH1‐positive or IL10‐positive cells were largely distributed in the osteochondral interface and the margin of the subchondral trabecular bone. In addition, a significant amount of EXPH1‐positive cells were noted in the TMJ discs in the UAC groups (Figures 4, 5, 6).

## 4 DISCUSSION

The present study was designed to identify some target genes in PBLs involved in TMJ OA‐like lesions induced by UAC, which was demonstrated to induce observable changes at the macro‐, micro‐ and molecular levels at 20 weeks of operation.^16^ First, a microarray analysis of samples from 20‐week UAC treatment rats and controls was detected. Candidate genes and proteins were selected when the expression fold differences between the PBLs of the UAC and control groups were over two times those of the top six upregulated GO categories. The three selected genes*, Egr1*, *Ephx1* and *Il10,* were verified by comparing real‐time PCR assay results between the UAC and control rats. Using Western blotting methods, the upregulation of EGR1, EPHX1 and IL10 was identified in the mandibular condylar cartilage and subchondral bone and that of EPHX1 was identified in the TMJ disc, all of which are tissues from TMJ; no upregulation was noted in the alveolar bone. None of the three molecules were expressed in the jaw muscle tissues detected by Western blotting assay (data not shown). Further analysis indicated that the increasing levels of EGR1 and EPHX1 in the UAC group occurred at 12 weeks in the mandibular condylar cartilage or subchondral bone. Increased EPHX1 expression also occurred in the discs at both 12 and 20 weeks, and an increase in IL10 protein expression was identified in the subchondral bone at 12 weeks but in the cartilage at 20 weeks. All three molecules were frequently detected at the osteochondral interface, as revealed by the IHC assay. Therefore, it is primarily the changes in the deep zone cartilage and the subchondral plate bone that contribute to the increased mRNA expression levels of *Egr1*, *Ephx1* and *Il10* in PBLs. These results agree with our recent report that the osteochondral interface could be a new diagnostic and therapeutic target.^16^

Biomarkers related to OA lesions are attractive.^21^ EGR1 is a transcription factor for growth and differentiation and serves as a regulator in several biological processes.^22^ EGR1 is involved in responding to mechanical stress in bone cells^23^, ^24^ and wound‐healing processes^25^ such as the healing of vessels, cartilages and bones.^22^, ^26^ It can be detected in the callus in multiple tissues, such as lung injuries, endothelial wounds, vascularised tissue and bone fractures.^25^ The depletion of *Egr1* in mice affects the structure of cortical bones.^25^ There is decreased bone mass in the limbs of adult *Egr1* ^−/−^mice.^27^ The expression of EGR1 increases after osteogenic differentiation with osteogenic differentiation medium.^22^ Currently, the EGR1 protein expression level was significantly higher in the condylar cartilage and subchondral bone of UAC rats than in the controls, but it was not elevated in the alveolar bone or TMJ discs. In addition, EGR1 was primarily distributed in the osteochondral interface and the margin of the subchondral trabecular bone. Osteoarthritis is suggested to be a traumatic degradative process in which there are activated maladaptive repair responses [http://oarsi.org/research/standardisation-osteoarthritis-definitions]. The upregulation of EGR1 in the TMJ cartilage and subchondral bone of the UAC rats and that identified in the PBLs is an indicator of an active response of the cartilage and subchondral bone tissues, particularly in the osteochondral interface and the margin of the subchondral trabecular bone; this action is a wound‐healing activity in response to the UAC trauma stimulation.

EPHX1 was reported to be linked to bone disease, as revealed by testing genomic DNA from myeloma patients using a custom‐built DNA single nucleotide polymorphism (SNP) chip.^28^ Soluble EPHX is a phase‐I xenobiotic metabolising enzyme. Its endogenous substrates include epoxyeicosatrienoic acids, which are involved in regulating blood pressure and inflammation and are metabolites of arachidonic acid.^29^, ^30^ Inhibitions of soluble EPHX provide protective effects against inflammatory alveolar bone loss.^29^ Presently, the EPHX1 expression level in the TMJ cartilage is 3.6 times higher in the UAC group at 12 weeks and 4.2 times higher at 20 weeks. These values are 3.3 and 5.1 times higher in the subchondral bone, respectively, and 1.6 and 2.0 times higher in the disc, respectively. The UAC‐induced TMJ OA‐like lesions worsen with time within 20 weeks.^16^ The timely enhanced increasing of EPHX1 protein expression in the UAC rat mandibular condylar cartilage, subchondral bone and TMJ disc indicates a progressive decrease in anti‐inflammation activity and a potential risk of cartilage and bone loss.

IL10 prevents the destruction of cartilage in rheumatoid arthritis and suppresses inflammatory reactions by blocking the signal transduction of cell surface receptors.^31^, ^32^ IL10 is a suppressor of monocyte/macrophage function and downregulates the synthesis of proinflammatory cytokines and chemokines, such as IL1, IL6 and TNF‐alpha,^32^ and the synthesis of nitric oxide, gelatinase and collagenase.^33^ The specific neutralisation of IL10 upregulates the synthesis of IL1 and TNF‐alpha.^34^ IL10 contributes to the maintenance of bone mass through the inhibition of osteoclastic bone resorption and the regulation of osteoblastic bone formation.^34^ TNF‐alpha, IL1 and IL6 are all proinflammatory cytokines that play important roles in the regulation of cartilage degradation and bone remodelling, particularly bone resorption.^32^ Osteoclasts can be directly activated by IL6; simultaneously, IL1 and TNF‐alpha upregulate RANKL on the surface of osteoblasts and/or stromal cells interacting with its cell surface receptor, RANK, on osteoclast precursors, resulting in increased numbers of osteoclasts.^32^ Our published data indicated that there was an increased expression of TNF‐alpha, together with the upregulated gene expression of IL1 and IL6, in the early stages of UAC‐treated rat and mouse TMJs.^17^, ^35^ The UAC‐stimulated TNF‐alpha accelerated the chondrocyte apoptosis induced by UAC via the death‐receptor pathway.^35^ However, in this long‐term study, according to the microarray assay, the downregulated fold changes of TNF‐alpha, IL1 and IL6 were 2.5, 1.4 and 1.5 times greater in the UAC_Gene group, respectively, than in the Con_Gene group. The time effects on the expression of the inflammatory factors remain unknown. By contrast, IL10 was upregulated up to 3.8‐fold higher in the UAC_Gene group than in the Con_Gene group, which can be verified by the mRNA expression in PBLs. Although protein levels in the subchondral bone were transiently increased, that of condyle cartilage was increased at 20 weeks, indicating the probable failure of an anti‐inflammation reaction and maintenance in subchondral bone but a subsequent start of those responses in condyle cartilage.

Although animal models were generally accepted for human disease investigation, humans are different from animals. The present outcomes must be verified by clinical data before being finalised. Considering the present indications that there are time‐ and multi‐tissue effects on the detected biomarkers, it is a challenge to enrol patients at different disease stages of OA without any other osteo‐cartilage problems, such as osteoporosis and periodontal problems, which are widespread in elderly people.

## 5 CONCLUSION

We used a microarray analysis to focus on the upregulation of three molecules in the PBLs of rats with dentally induced TMJ OA‐like lesions, including *Egr1*, which is related to tissue wound‐healing processes, *Ephx1,* which is related to inflammatory responses and tissue destruction, and *Il10*, which plays a role in preventing the destruction of cartilage and suppressing inflammatory reactions. The three gene‐encoded proteins are predominantly noticed in the mandibular condylar cartilage and subchondral plate bone region. OA is generally considered a local joint problem. The present results, for the first time, identified the implication of the biological markers EGR1, EPHX1 and IL10 in PBLs that are involved in joint tissue damage and repair and provide new insights for the clinical detection and evaluation of TMJ OA lesions. Further clinical investigation is necessary.

## CONFLICT OF INTEREST

All authors declare no potential conflicts of interest with respect to the authorship and/or publication of this article.
