# Supplementary material for: Molecular changes in peripheral blood involving osteoarthritic joint remodelling
Source: J Oral Rehabil. 2019 May 11;46(9):820–7. doi: 10.1111/joor.12810 (PMC6851883; doi:10.1111/joor.12810)
Supplement: Supplementary file 1 [file JOOR-46-820-s001.docx]

**SUPPLEMENT METHODS.**

**Microarray and data analysis**

The Agilent Rat mRNA Array was designed with eight identical arrays per slide (8 x 60K format), with each array containing probes interrogating about 30,003 Entrez Gene RNAs. The purity and concentration of RNA were determined from OD260/280 readings using spectrophotometer (NanoDrop ND-1000). RNA integrity was determined by 1% formaldehyde denaturing gel electrophoresis. cDNA labeled with a fluorescent dye (Cy5 or Cy3-dCTP) was produced by Eberwine’s linear RNA amplification method and subsequent enzymatic reaction using CapitalBio cRNA Amplification and Labeling Kit (CapitalBio, Beijing, China) for producing higher yields of labeled cDNA.

The array data were analyzed for data summarization, normalization and quality control by using the GeneSpring software V13 (Agilent). To select the differentially expressed genes, we used threshold values of ≥ 2 and ≤ −2-fold change and t-test *P* value of 0.05. The data was Log2 transformed and median centered by genes using the Adjust Data function of CLUSTER 3.0 software then further analyzed with hierarchical clustering with average linkage. Finally, we performed tree visualization by using Java Treeview (Stanford University School of Medicine, Stanford, CA, USA).
